# Supplementary material for: Structure-based design of a strain transcending AMA1-RON2L malaria vaccine
Source: Nat Commun. 2023 Sep 2;14:5345. doi: 10.1038/s41467-023-40878-7 (PMC10475129; doi:10.1038/s41467-023-40878-7)
Supplement: Supplementary file 1 — Supplementary Information [file 41467_2023_40878_MOESM1_ESM.pdf]

Supplementary Information for:

Structure-based design of a strain transcending AMA1-RON2L malaria vaccine

Palak N. Patel[1], Thayne H. Dickey[1], Ababacar Diouf[2], Nichole D. Salinas[1], Holly McAleese[3], Tarik Ouahes[3], Carole A. Long[2], Kazutoyo Miura[2], Lynn E. Lambert[3], Niraj H. Tolia[1]*

[1]Host-Pathogen Interactions and Structural Vaccinology Section, Laboratory of Malaria Immunology and Vaccinology, National Institute of Allergy and Infectious Diseases, National Institutes of Health, Bethesda, MD, USA

[2]Laboratory of Malaria and Vector Research, National Institute of Allergy and Infectious Diseases, National Institutes of Health, Rockville, MD, USA

[3] Vaccine Development Unit, Laboratory of Malaria Immunology and Vaccinology, National Institute of Allergy and Infectious Diseases, National Institutes of Health, Bethesda, MD, USA

*Corresponding author:

niraj.tolia@nih.gov

9000 Rockville Pike, Building 29B, 4NN08

Bethesda, MD, 20892

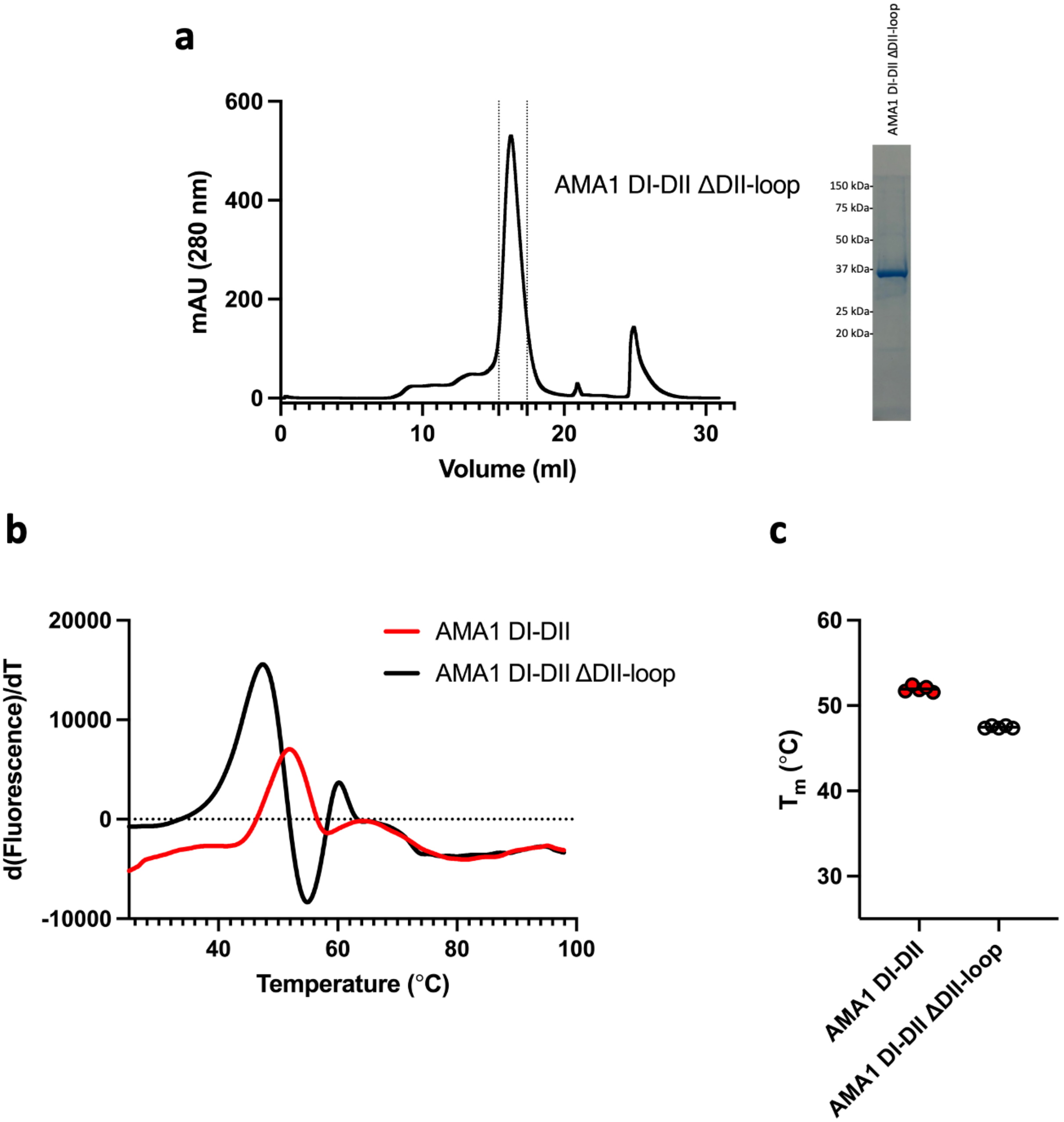

Supplementary Fig. 1: Purification and characterization of the AMA1 DI-DII ΔDII-loop. a Size exclusion chromatography (SEC) profile of the AMA1 DI-DII ΔDII-loop. The peak for the AMA1 DI-DII ΔDII-loop is shown between two dotted lines. Inset shows Coomassie Brilliant Blue-stained SDS-PAGE gel for AMA1 DI-DII ΔDII-loop (35.4-kDa) under reducing condition. **b** Differential scanning fluorimetry indicated that deletion of DII loop does not improve the stability of engineered AMA1 DI-DII. **c** Tm from five independent measurements. Bar represents mean. Source data are provided as a Source data file.

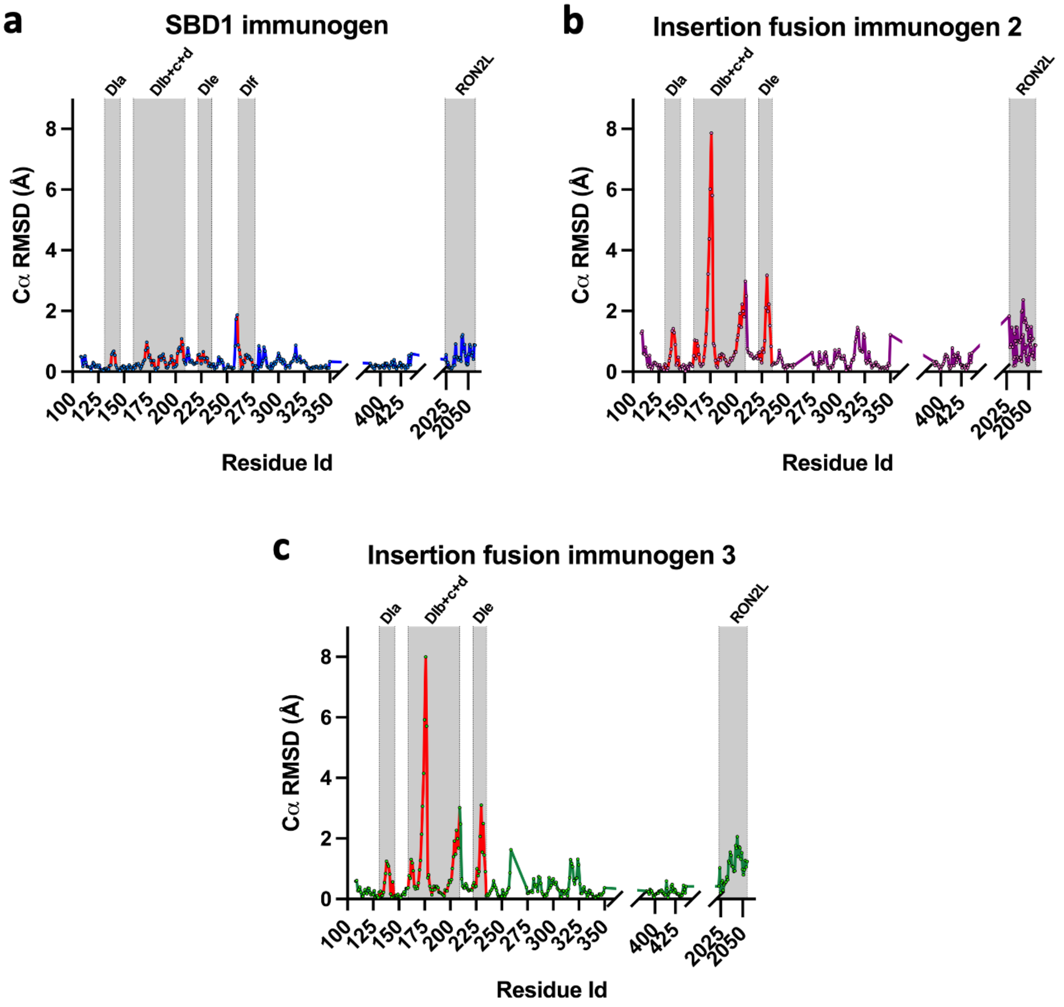

Supplementary Fig. 2: SBD1 immunogen possesses the greatest structural similarity to the AMA1-RON2L complex. The root-mean-square deviation (RMSD) for the Cα atom of each residue in the **a** SBD1 immunogen, **b** Insertion fusion immunogen 2, and **c** Insertion fusion immunogen 3, when aligned to the AMA1 DI-DII-RON2L complex (PDB ID: 3zwz, https://www.rcsb.org/structure/3ZWZ). The residues are labeled based on the wildtype AMA1 and RON2 sequence numbering. The domain I loops are indicated by red lines and silver shades beneath the corresponding data points. Source data are provided as a Source data file.

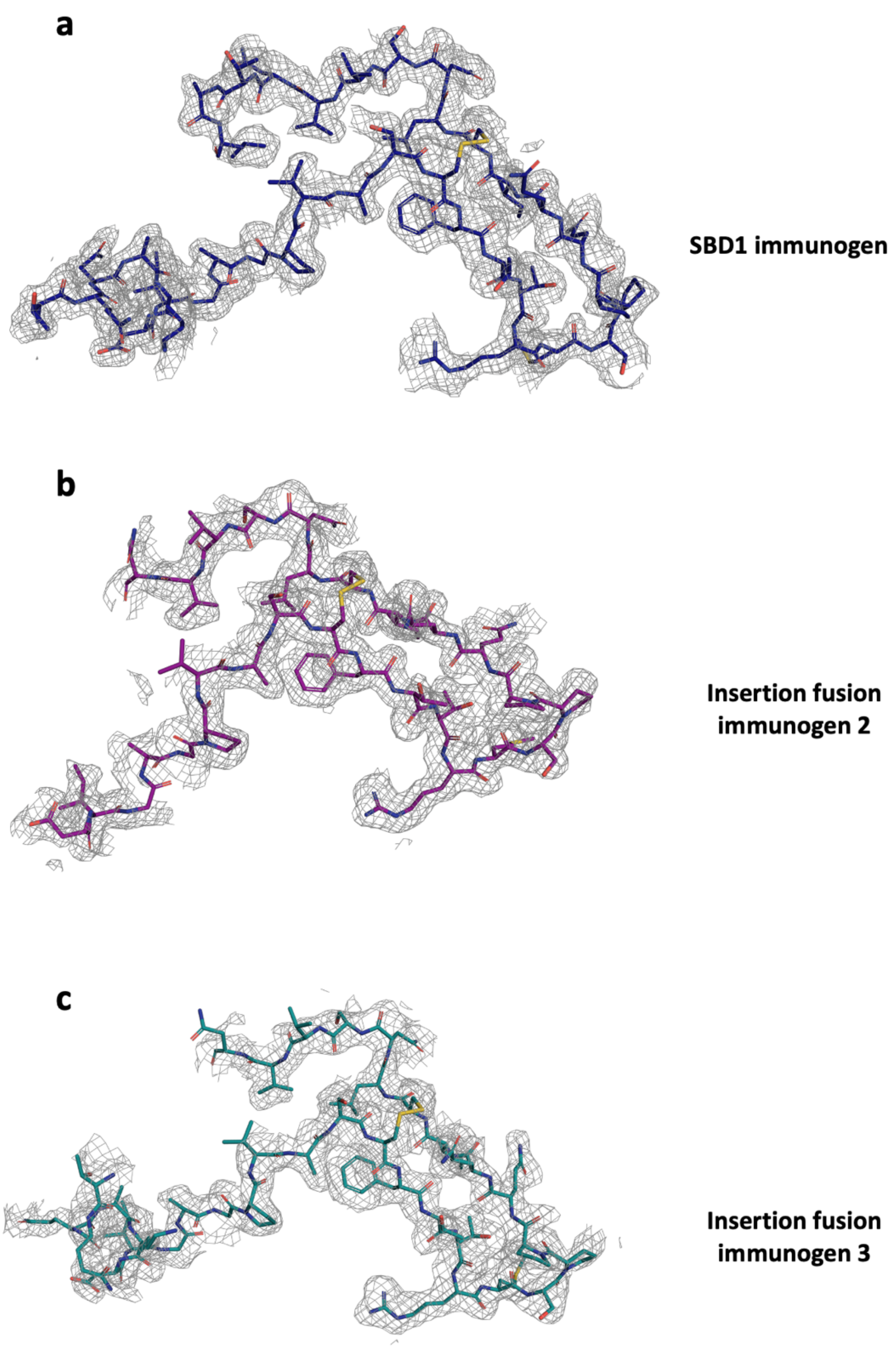

Supplementary Fig. 3: RON2L of single-component immunogens shows a disulfide-anchored U-shaped conformation in the hydrophobic groove of AMA1. Electron density for the RON2L component of **a** SBD1 immunogen, **b** Insertion fusion immunogen 2, and **c** Insertion fusion immunogen 3 from a Polder map (gray mesh) contoured at 1.0 σ level (1.43 rmsd).

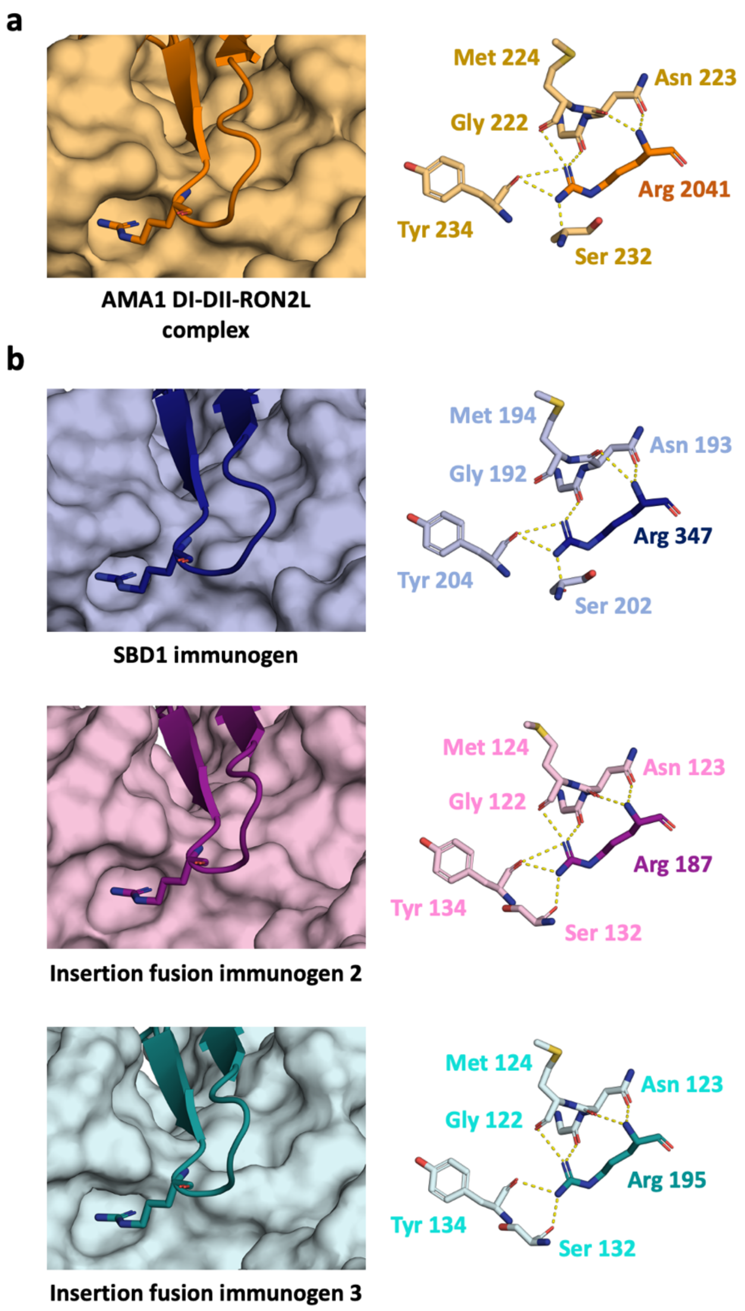

Supplementary Fig. 4: An influential residue, Arg2041, is located at the tip of the β-hairpin and with its guanidyl group is adequately positioned within the preformed pocket of AMA1. As in **a** AMA1 DI-DII-RON2L complex structure (PDB ID: 3zwz, https://www.rcsb.org/structure/3ZWZ), the Arg residue of RON2L of **b** all three single-component immunogens fits snugly into a deep pocket in the surface of AMA1. The complex network of hydrogen bonds stabilizes this structure.

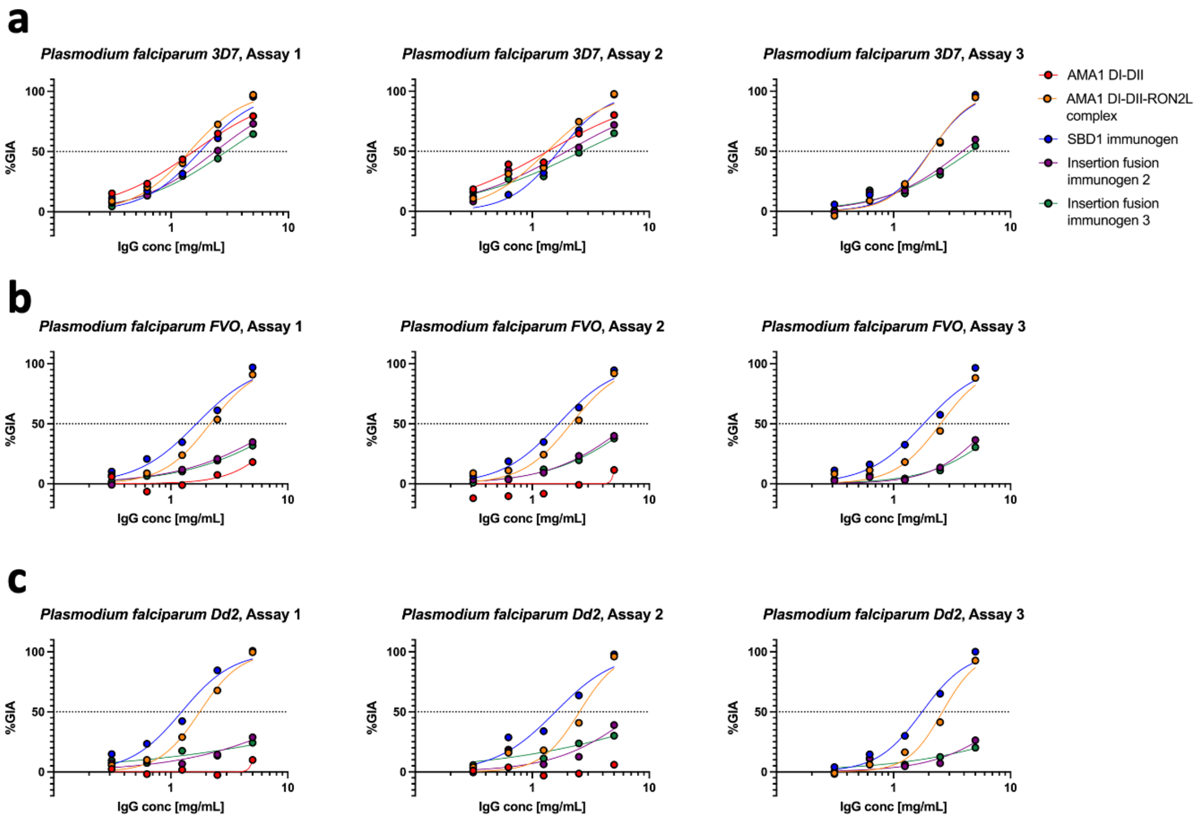

Supplementary Fig. 5: *In vitro* GIA dilution series of pooled purified IgG from each group at day 63 against *Plasmodium falciparum* a *3D7* b *FVO* c *Dd2*. Data from individual assays are shown. IC_{50} values were determined by interpolation after fitting data to a four-parameter dose-response curve. IgG from AMA1 DI-DII IgG group was not tested in Assay 3 for each strain. Source data are provided as a Source data file.

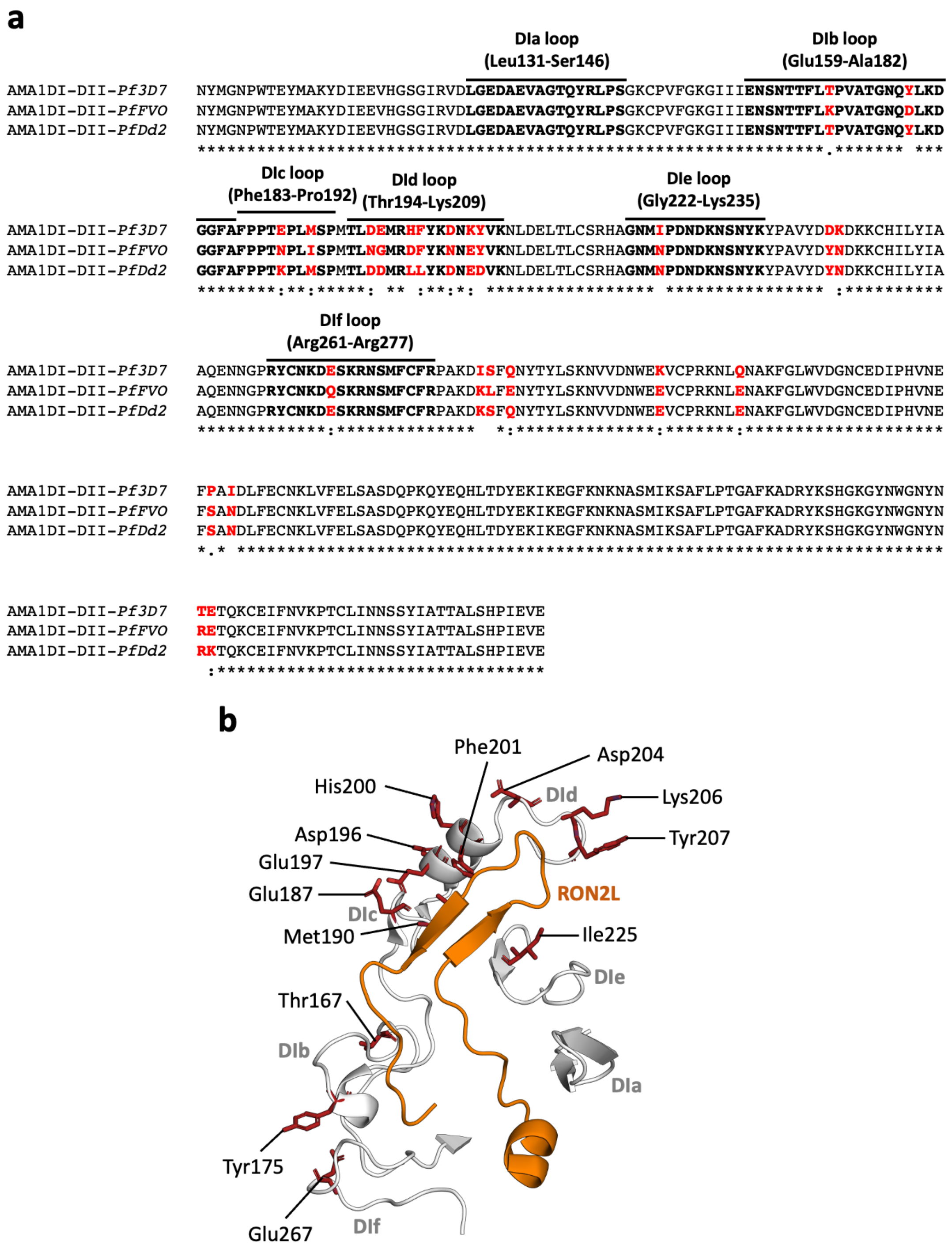

Supplementary Fig. 6: Immunization with the SBD1 immunogen induces higher levels of broadly neutralizing antibodies potentially by targeting new functional regions on AMA1 including conserved domain I loops. a Sequence alignment of AMA1 DI-DII from *Plasmodium falciparum 3D7*, *FVO*, and *Dd2* strains. Polymorphic residues, which vary between the strains, are marked in bold red. Residues within the

AMA1 domain I loops surrounding the RON2L binding site, in the presence of RON2L, are marked in bold and labeled. The multiple sequence alignments were generated using the Clustal Omega/TCoffee. **b** Polymorphic residues mapped onto the AMA1 domain I loops surrounding the RON2L binding site in the presence of RON2L (PDB ID: 3zwz, https://www.rcsb.org/structure/3ZWZ). The polymorphic residues are depicted as red sticks, indicating their positions within the AMA1 domain I loops. The AMA1 loops are represented as a white cartoon (labeled DIa, DIb, DIc, DId, DIe, and DIf loops). The RON2L is depicted as an orange cartoon.

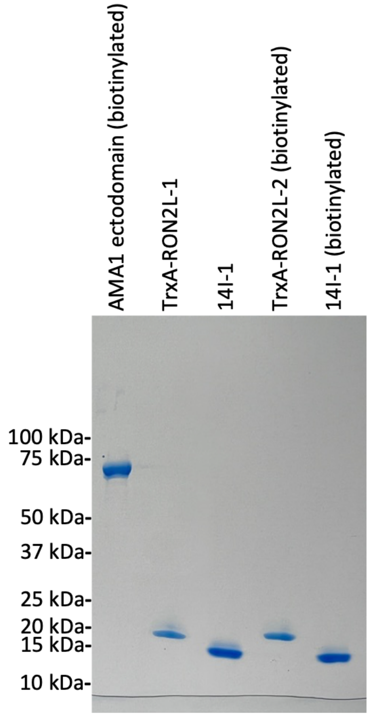

Supplementary Fig. 7: Coomassie Brilliant Blue-stained SDS-PAGE gel for AMA1 ectodomain (biotinylated, 63.8-kDa), TrxA-RON2L-1 (18.1-kDa), IgNAR 14I-1 (14.2-kDa), TrxA-RON2L-2 (biotinylated, 20.2-kDa) and IgNAR 14I-1 (biotinylated, 16.7-kDa) under reducing condition. Source data are provided as a Source data file.

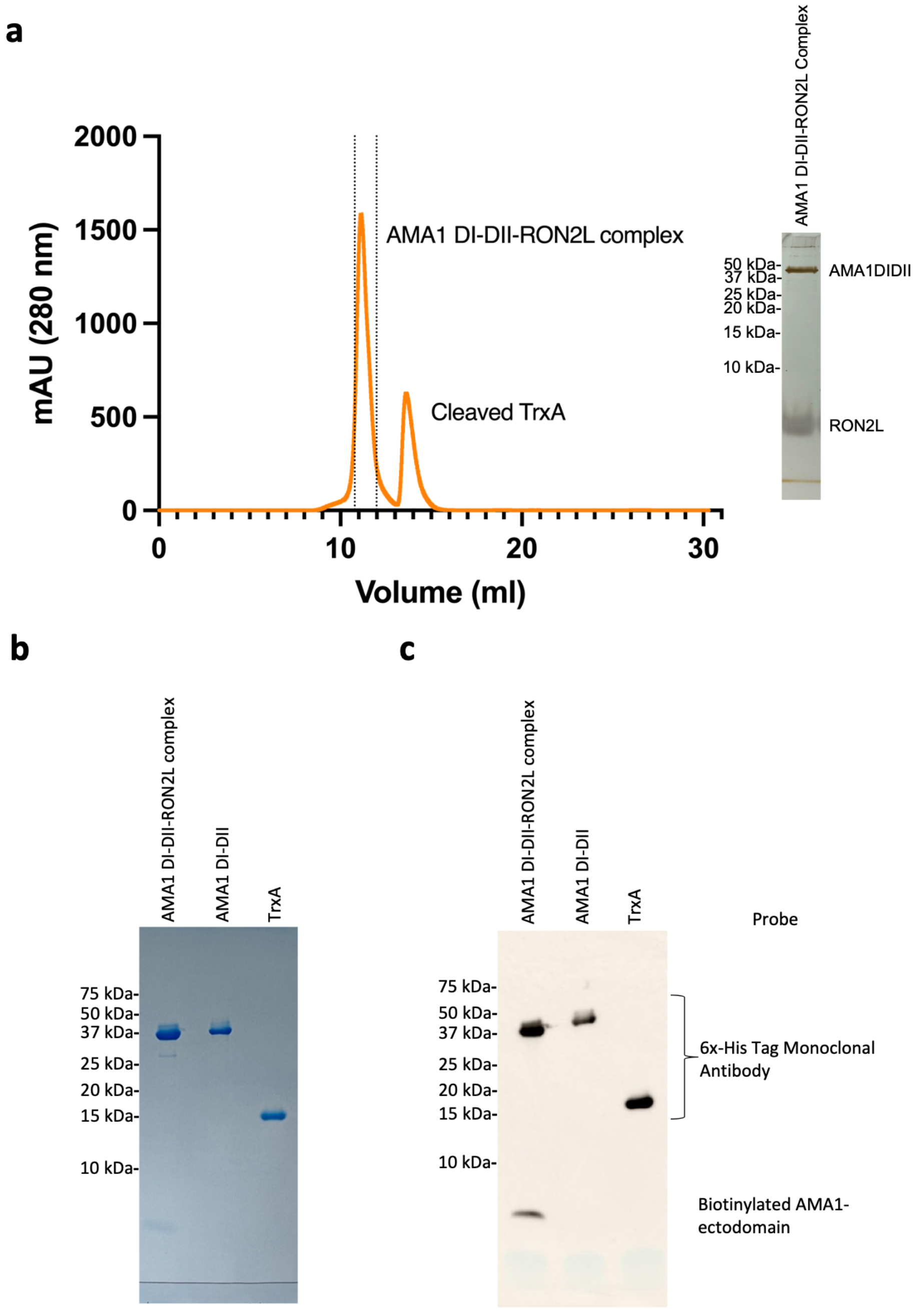

Supplementary Fig. 8: Purification and characterization of the AMA1 DI-DII-RON2L complex. a Size exclusion chromatography (SEC) profile of the AMA1 DI-DII-RON2L complex. The peak for the AMA1 DI-DII-RON2L complex is shown between two dotted lines. A second peak corresponds to cleaved TrxA (6x-His tagged). Inset shows silver-stained SDS-PAGE gel for AMA1 DI-DII (39.2-kDa) and RON2L (4.2-kDa) components of complex under reducing condition. **b** Coomassie Brilliant Blue-stained SDS-PAGE gel for AMA1 DI-DII and RON2L components of complex under non-reducing condition. **c**

Western blot for the AMA1 DI-DII and RON2L components of complex is depicted, along with the primary probe used. AMA1 DI-DII (39.2-kDa) and TrxA (14.73-kDa) were used as controls under non-reducing condition. Source data are provided as a Source data file.

Supplementary Table 1: Sequences of immunogens and constructs used in the study after signal peptide cleavage. Cloning scars, tags, and linkers are shown in lowercase.

Name	Sequence
**Native AMA1 DI-DII**	etgNYMGNPWTEYMAKYDIEEVHGSGIRVDLGEDAEVAGTQYRLPSGKCPVFGKGIIIENSNTAFLTPVATGNQYLKDGGFAFPPTEPLMSPMTLDEMRHFYKDNKYVKNLDELTLCSRHAGNMIPDNDKNSNYKYPAVYDDKDKKCHILYIAAQENNGPRYCNKDESKRNSMFCFRPAKDISFQNYAYLSKNVVDNWEKVCPRKNLQNAKFGLWVDGNCEDIPHVNEFPAIDLFECNKLVFELSASDQPKQYEQHLTDYEKIKEGFKNKNAAMIKSAFLPTGAFKADRYKSHGKGYNWGNYNTETQKCEIFNVKPTCLINNAAYIATTALSHPIEVEgtkhhhhhhh
**AMA1 DI-DII ΔDII-loop**	etgNYMGNPWTEYMAKYDIEEVHGSGIRVDLGEDAEVAGTQYRLPSGKCPVFGKGIIIENSNTAFLTPVATGNQYLKDGGFAFPPTEPLMSPMTLDEMRHFYKDNKYVKNLDELTLCSRHAGNMIPDNDKNSNYKYPAVYDDKDKKCHILYIAAQENNGPRYCNKDESKRNSMFCFRPAKDISFQNYAYLSKNVVDNWEKVCPRKNLQNAKFGLWVDGNCEDIPHVNEFPAIDLFECNKLVFELSASDQPGGSGYKSHGKGYNWGNYNTETQKCEIFNVKPTCLINNAAYIATTALSHPIEVEgtkhhhhhh
**SBD1 immunogen**	etgKADRYKSHGKGYNWGNYNTETQKCEIFNVKPTCLINNAAYIATTALSHPIEVEggggsggggsggggsggggsNYMGNPWTEYMAKYDIEEVHGSGIRVDLGEDAEVAGTQYRLPSGKCPVFGKGIIIENSNTAFLTPVATGNQYLKDGGFAFPPTEPLMSPMTLDEMRHFYKDNKYVKNLDELTLCSRHAGNMIPDNDKNSNYKYPAVYDDKDKKCHILYIAAQENNGPRYCNKDESKRNSMFCFRPAKDISFQNYAYLSKNVVDNWEKVCPRKNLQNAKFGLWVDGNCEDIPHVNEFPAIDLFECNKLVFELSASDQPKQYEQHLTTQQAKDIGAGPVASCFTTRMSPPQQICLNSVVNTALSgtkhhhhhh
**Insertion fusion immunogen 2**	etgNYMGNPWTEYMAKYDIEEVHGSGIRVDLGEDAEVAGTQYRLPSGKCPVFGKGIIIENSNTAFLTPVATGNQYLKDGGFAFPPTEPLMSPMTLDEMRHFYKDNKYVKNLDELTLCSRHAGNMIPDNDKNSNYKYPAVYDDKDKKCHILYIAAQENNGggggsgsDITQQAKDIGAGPVASCFTTRMSPPQQICLNSVVNTALSggsgMFCFRPAKDISFQNYAYLSKNVVDNWEKVCPRKNLQNAKFGLWVDGNCEDIPHVNEFPAIDLFECNKLVFELSASDQPggsgYKSHGKGYNWGNYNTETQKCEIFNVKPTCLINNAAYIATTALSHPIEVEgtkhhhhhh
**Insertion fusion immunogen 3**	etgNYMGNPWTEYMAKYDIEEVHGSGIRVDLGEDAEVAGTQYRLPSGKCPVFGKGIIIENSNTAFLTPVATGNQYLKDGGFAFPPTEPLMSPMTLDEMRHFYKDNKYVKNLDELTLCSRHAGNMIPDNDKNSNYKYPAVYDDKDKKCHILYIAAQENNGPRYCNGgggsggggsDITQQAKDIGAGPVASCFTTRMSPPQQICLNSVVNTALSggsgMFCFRPAKDISFQNYAYLSKNVVDNWEKVCPRKNLQNAKFGLWVDGNCEDIPHVNEFPAIDLFECNKLVFELSASDQPggsgYKSHGKGYNWGNYNTETQKCEIFNVKPTCLINNAAYIATTALSHPIEVEgtkhhhhhh
**AMA1 ectodomain**	etgQNYWEHPYQNSDVYRPINEHREHPKEYEYPLHQEHTYQQEDSGEDENTLQHAYPIDHEGAEPAPQEQNLFSSIEIVERSNYMGNPWTEYMAKYDIEEVHGSGIRVDLGEDAEVAGTQYRLPSGKCPVFGKGIIIENSNTTFLTPVATGNQYLKDGGFAFPPTEPLMSPMTLDEMRHFYKDNKYVKNLDELTLCSRHAGNMIPDNDKNSNYKYPAVYDDKDKKCHILYIAAQENNGPRYCNKDESKRNSMFCFRPAKDISFQNYTYLSKNVVDNWEKVCPRKNLQNAKFGLWVDGNCEDIPHVNEFPAIDLFECNKLVFELSASDQPKQYEQHLTDYEKIKEGFKNKNASMIKSAFLPTGAFKADRYKSHGKGYNWGNYNTETQKCEIFNVKPTCLINNSSYIATTALSHPIEVENNFPCSLYKDEIMKEIERESKRIKLNDNDDEGNKKIIAPRIFISDDKDSLKCPCDPEMVSNSTCRFFVCKCVERRAEVTSNNEVVVKEEYKDEYADIPEHKPTgtggsggsglndifeaqkiewhegrtkhhhhhh
**TrxA-RON2L-1**	etghhhhhhMSDKIIHLTDDSFDTDVLKADGAILVDFWAEWCGPCKMIAPILDEIADEYQGKLTVAKLNIDQNPGTAPKYGIRGIPTLLLFKNGEVAATKVGALSKGQLKEFLDANLAgsgslevlfqgpDITQQAKDIGAGPVASCFTTRMSPPQQICLNSVVNTALS
**TrxA-RON2L-2**	etghhhhhhMSDKIIHLTDDSFDTDVLKADGAILVDFWAEWCGPCKMIAPILDEIADEYQGKLTVAKLNIDQNPGTAPKYGIRGIPTLLLFKNGEVAATKVGALSKGQLKEFLDANLAGSGSLEVLFQGPDITQQAKDIGAGPVASCFTTRMSPPQQICLNSVVNTALSGSGSglndifeaqkiewhe
**TrxA**	etghhhhhhMSDKIIHLTDDSFDTDVLKADGAILVDFWAEWCGPCKMIAPILDEIADEYQGKLTVAKLNIDQNPGTAPKYGIRGIPTLLLFKNGEVAATKVGALSKGQLKEFLDANLAGSGSglndifeaqkiewhe
**14I-1**	etgAWVDQTPRTATKETGESLTINCVLRDASLELKDTGWYRTKLGSTNEQSISIGGRYVETVNKGSKSFSLRISDLRVEDSGTYKCQAFYSLPLRDYNYALLFRGEKGAGTALTVKAAAgtkhhhhhh
**14I-1 (biotinylated)**	etgAWVDQTPRTATKETGESLTINCVLRDASLELKDTGWYRTKLGSTNEQSISIGGRYVETVNKGSKSFSLRISDLRVEDSGTYKCQAFYSLPLRDYNYALLFRGEKGAGTALTVKAAAgtggsggsglndifeaqkiewhegrtkhhhhhh

Supplementary Table 2: Contact residues between RON2L and AMA1 residues for SBD1 immunogen.

**Residues in RON2L**	**Residues in RON2L (Numbered according to PDB ID:3ZWZ)**	**Interface with AMA1 (PDB ID: 3ZWZ)**	**Residues in RON2L (SBD1 immunogen)**	**Interface with AMA1 (SBD1 immunogen)**	**Interface with AMA1 (SBD1 immunogen, numbered according to PDB ID:3ZWZ)**
**Thr3**	**Thr2023**	Leu131	**Thr329**	Leu101	Leu131
**Gln4**	**Gln2024**	Val137	**Gln330**	Val107	Val137
**Gln5**	**Gln2025**	Thr140	**Gln331**	Thr110	Thr140
**Ala6**	**Ala2026**	Gln141	**Ala332**	Gln111	Gln141
**Lys7**	**Lys2027**	Tyr142	**Lys333**	Tyr112	Tyr142
**Asp8**	**Asp2028**	Arg143	**Asp334**	Arg113	Arg143
**Ile9**	**Ile2029**	Val169	**Ile335**	Val139	Val169
**Gly10**	**Gly2030**	Ala170	**Gly336**	Ala140	Ala170
**Ala11**	**Ala2031**	Thr171	**Ala337**	Thr141	Thr171
**Gly12**	**Gly2032**	Gly172	**Gly338**	Gly142	Gly172
**Pro13**	**Pro2033**	Gln174	**Pro339**	Gln144	Gln174
**Val14**	**Val2034**	Leu176	**Val340**	Tyr145	Tyr175
**Ala15**	**Ala2035**	Phe183	**Ala341**	Leu146	Leu176
**Ser16**	**Ser2036**	Pro184	**Ser342**	Phe153	Phe183
**Cys17**	**Cys2037**	Pro185	**Cys343**	Pro154	Pro184
**Phe18**	**Phe2038**	Thr186	**Phe344**	Pro155	Pro185
**Thr19**	**Thr2039**	Glu187	**Thr345**	Thr156	Thr186
**Thr20**	**Thr2040**	Pro188	**Thr346**	Glu157	Glu187
**Arg21**	**Arg2041**	Met190	**Arg347**	Pro158	Pro188
**Met22**	**Met2042**	Phe201	**Met348**	Met160	Met190
**Ser23**	**Ser2043**	Tyr202	**Ser349**	Phe171	Phe201
**Pro24**	**Pro2044**	Asn205	**Pro350**	Tyr172	Tyr202
**Pro25**	**Pro2045**	Val208	**Pro351**	Asn175	Asn205
**Gln26**	**Gln2046**	Arg219	**Gln352**	Val178	Val208
**Gln27**	**Gln2047**	Gly222	**Gln353**	Arg189	Arg219
**Ile28**	**Ile2048**	Asn223	**Ile354**	Gly192	Gly222
**Cys29**	**Cys2049**	Met224	**Cys355**	Asn193	Asn223
**Leu30**	**Leu2050**	Ile225	**Leu356**	Met194	Met224
**Asn31**	**Asn2051**	Pro226	**Asn357**	Ile195	Ile225
**Ser32**	**Ser2052**	Asp227	**Ser358**	Pro196	Pro226
**Val33**	**Val2053**	Asn228	**Val359**	Asp197	Asp227
**Val34**	**Val2054**	Ser232	**Val360**	Asn198	Asn228
**Asn35**	**Asn2055**	Tyr234	**Asn361**	Ser202	Ser232
**Thr36**	**Thr2056**	Lys235	**Thr362**	Tyr204	Tyr234
**Ala37**	**Ala2057**	Tyr236	**Ala363**	Lys205	Lys235
**Leu38**	**Leu2058**	Tyr251	**Leu364**	Tyr206	Tyr236
		Ile252		Tyr221	Tyr251
		Ala253		Ile222	Ile252
		Ala254		Ala223	Ala253
		Gln255		Ala224	Ala254
		Glu256		Gln225	Gln255
		Asn257		Glu226	Glu256
		Tyr262		Asn227	Asn257
		Asn271		Tyr232	Tyr262
		Met273		Asn241	Asn271
		Phe274		Met243	Met273
		Phe276		Phe244	Phe274
		Gln349		Phe246	Phe276
		Pro350		Gln319	Gln349
		Arg389		Pro320	Pro350
		Tyr390		Gln325	Gln355
				Leu327	Leu357
				Thr328	Thr358
				Arg4	Arg389
				Tyr5	Tyr390

A red box indicates residues present at the interface of the AMA1 DI-DII-RON2L complex (PDB: 3zwz, https://www.rcsb.org/structure/3ZWZ) but not in the SBD1 immunogen, while a green box indicates residues present at the interface of the SBD1 immunogen but absent in the complex.

Supplementary Table 3: Contact residues between RON2L and AMA1 residues for Insertion fusion immunogen 2.

Residues in RON2L	Residues in RON2L (Numbered according to PDB ID:3ZWZ)	Interface with AMA1 (PDB ID: 3ZWZ)	Residues in RON2L (Insertion fusion immunogen 2)	Interface with AMA1 (Insertion fusion immunogen 2)	Interface with AMA1 (Insertion fusion immunogen 2, numbered according to PDB ID:3ZWZ)
**Asp8**	**Asp2028**	Leu131	**Asp174**	Leu31	Leu131
**Ile9**	**Ile2029**	Val137	**Ile175**	Val37	Val137
**Gly10**	**Gly2030**	Tyr142	**Gly176**	Tyr42	Tyr142
**Ala11**	**Ala2031**	Arg143	**Ala177**	Arg43	Arg143
**Gly12**	**Gly2032**	Val169	**Gly178**	Val69	Val169
**Pro13**	**Pro2033**	Ala170	**Pro179**	Ala70	Ala170
**Val14**	**Val2034**	Thr171	**Val180**	Thr71	Thr171
**Ala15**	**Ala2035**	Gly172	**Ala181**	Gly72	Gly172
**Ser16**	**Ser2036**	Gln174	**Ser182**	Asn73	Asn173
**Cys17**	**Cys2037**	Leu176	**Cys183**	Lys77	Lys177
**Phe18**	**Phe2038**	Phe183	**Phe184**	Phe83	Phe183
**Thr19**	**Thr2039**	Pro184	**Thr185**	Pro84	Pro184
**Thr20**	**Thr2040**	Pro185	**Thr186**	Pro85	Pro185
**Arg21**	**Arg2041**	Thr186	**Arg187**	Thr86	Thr186
**Met22**	**Met2042**	Glu187	**Met188**	Glu87	Glu187
**Ser23**	**Ser2043**	Pro188	**Ser189**	Pro88	Pro188
**Pro24**	**Pro2044**	Met190	**Pro190**	Met90	Met190
**Pro25**	**Pro2045**	Phe201	**Pro191**	Phe101	Phe201
**Gln26**	**Gln2046**	Tyr202	**Gln192**	Tyr102	Tyr202
**Gln27**	**Gln2047**	Asn205	**Gln193**	Asp104	Asp204
**Ile28**	**Ile2048**	Val208	**Ile194**	Asn105	Asn205
**Cys29**	**Cys2049**	Arg219	**Cys195**	Gly122	Gly222
**Leu30**	**Leu2050**	Gly222	**Leu196**	Asn123	Asn223
**Asn31**	**Asn2051**	Asn223	**Asn197**	Met124	Met224
**Ser32**	**Ser2052**	Met224	**Ser198**	Ile125	Ile225
**Val33**	**Val2053**	Ile225	**Val199**	Pro126	Pro226
**Val34**	**Val2054**	Pro226	**Val200**	Asp127	Asp227
**Asn35**	**Asn2055**	Asp227	**Asn201**	Asn128	Asn228
		Asn228		Lys130	Lys230
		Ser232		Ser132	Ser232
		Tyr234		Asn133	Asn233
		Lys235		Tyr134	Tyr234
		Tyr236		Lys135	Lys235
		Tyr251		Tyr136	Tyr236
		Ile252		Tyr151	Tyr251
		Ala253		Ile152	Ile252
		Ala254		Ala153	Ala253
		Gln255		Ala154	Ala254
		Glu256		Gln155	Gln255
		Asn257		Glu156	Glu256
		Tyr262		Asn157	Asn257
		Asn271		Phe213	Phe276
		Phe276		Gln286	Gln349
		Gln349		Pro287	Pro350
		Pro350			
		Tyr390			

A red box indicates residues present at the interface of the AMA1 DI-DII-RON2L complex (PDB: 3zwz, https://www.rcsb.org/structure/3ZWZ) but not in the Insertion fusion immunogen 2, while a green box indicates residues present at the interface of the Insertion fusion immunogen 2 but absent in the complex.

Supplementary Table 4: Contact residues between RON2L and AMA1 residues for Insertion fusion immunogen 3.

Residues in RON2L	Residues in RON2L (Numbered according to PDB ID:3ZWZ)	Interface with AMA1 (PDB ID: 3ZWZ)	Residues in RON2L (Insertion fusion immunogen 3)	Interface with AMA1 (Insertion fusion immunogen 3)	Interface with AMA1 (Insertion fusion immunogen 3, numbered according to PDB ID:3ZWZ)
**Thr3**	**Thr2023**	Leu131	**Thr177**	Leu31	Leu131
**Gln4**	**Gln2024**	Val137	**Gln178**	Val37	Val137
**Gln5**	**Gln2025**	Thr140	**Gln179**	Thr40	Thr140
**Ala6**	**Ala2026**	Gln141	**Ala180**	Gln41	Gln141
**Lys7**	**Lys2027**	Tyr142	**Lys181**	Tyr42	Tyr142
**Asp8**	**Asp2028**	Arg143	**Asp182**	Arg43	Arg143
**Ile9**	**Ile2029**	Val169	**Ile183**	Val69	Val169
**Gly10**	**Gly2030**	Ala170	**Gly184**	Ala70	Ala170
**Ala11**	**Ala2031**	Thr171	**Ala185**	Thr71	Thr171
**Gly12**	**Gly2032**	Gly172	**Gly186**	Gly72	Gly172
**Pro13**	**Pro2033**	Gln174	**Pro187**	Asn73	Asn173
**Val14**	**Val2034**	Leu176	**Val188**	Lys77	Lys177
**Ala15**	**Ala2035**	Phe183	**Ala189**	Phe83	Phe183
**Ser16**	**Ser2036**	Pro184	**Ser190**	Pro84	Pro184
**Cys17**	**Cys2037**	Pro185	**Cys191**	Pro85	Pro185
**Phe18**	**Phe2038**	Thr186	**Phe192**	Thr86	Thr186
**Thr19**	**Thr2039**	Glu187	**Thr193**	Glu87	Glu187
**Thr20**	**Thr2040**	Pro188	**Thr194**	Pro88	Pro188
**Arg21**	**Arg2041**	Met190	**Arg195**	Met90	Met190
**Met22**	**Met2042**	Phe201	**Met196**	Phe101	Phe201
**Ser23**	**Ser2043**	Tyr202	**Ser197**	Tyr102	Tyr202
**Pro24**	**Pro2044**	Asn205	**Pro198**	Asp104	Asp204
**Pro25**	**Pro2045**	Val208	**Pro199**	Asn105	Asn205
**Gln26**	**Gln2046**	Arg219	**Gln200**	Gly122	Gly222
**Gln27**	**Gln2047**	Gly222	**Gln201**	Asn123	Asn223
**Ile28**	**Ile2048**	Asn223	**Ile202**	Met124	Met224
**Cys29**	**Cys2049**	Met224	**Cys203**	Ile125	Ile225
**Leu30**	**Leu2050**	Ile225	**Leu204**	Pro126	Pro226
**Asn31**	**Asn2051**	Pro226	**Asn205**	Asp127	Asp227
**Ser32**	**Ser2052**	Asp227	**Ser206**	Asn128	Asn228
**Val33**	**Val2053**	Asn228	**Val207**	Lys130	Lys230
**Val34**	**Val2054**	Ser232	**Val208**	Ser132	Ser232
**Asn35**	**Asn2055**	Tyr234	**Asn209**	Asn133	Asn233
		Lys235		Tyr134	Tyr234
		Tyr236		Lys135	Lys235
		Tyr251		Tyr136	Tyr236
		Ile252		Tyr151	Tyr251
		Ala253		Ile152	Ile252
		Ala254		Ala153	Ala253
		Gln255		Ala154	Ala254
		Glu256		Gln155	Gln255
		Asn257		Glu156	Glu256
		Tyr262		Asn157	Asn257
		Asn271		Phe221	Phe276
		Phe276		Gln294	Gln349
		Gln349		Pro295	Pro350
		Pro350		Tyr300	Tyr390
		Arg389			
		Tyr390			

A red box indicates residues present at the interface of the AMA1 DI-DII-RON2L complex (PDB: 3zwz, https://www.rcsb.org/structure/3ZWZ) but not in the fusion 3,

while a green box indicates residues present at the interface of the fusion 3 but absent in the complex.
