# Supplementary material for: Structure-based design of a strain transcending AMA1-RON2L malaria vaccine
Source: Nat Commun. 2023 Sep 2;14:5345. doi: 10.1038/s41467-023-40878-7 (PMC10475129; doi:10.1038/s41467-023-40878-7)
Supplement: Supplementary file 3 — Reporting Summary [file 41467_2023_40878_MOESM3_ESM.pdf]

nature portfolio

Corresponding author(s): Niraj Tolia

Last updated by author(s): Mar 24, 2023

# Reporting Summary

Nature Portfolio wishes to improve the reproducibility of the work that we publish. This form provides structure for consistency and transparency in reporting. For further information on Nature Portfolio policies, see our Editorial Policies and the Editorial Policy Checklist.

## Statistics

For all statistical analyses, confirm that the following items are present in the figure legend, table legend, main text, or Methods section.

| n/a | Confirmed | |
|---|---|---|
| ☐ | ☒ | The exact sample size (*n*) for each experimental group/condition, given as a discrete number and unit of measurement |
| ☐ | ☒ | A statement on whether measurements were taken from distinct samples or whether the same sample was measured repeatedly |
| ☐ | ☒ | The statistical test(s) used AND whether they are one- or two-sided<br>*Only common tests should be described solely by name; describe more complex techniques in the Methods section.* |
| ☒ | ☐ | A description of all covariates tested |
| ☐ | ☒ | A description of any assumptions or corrections, such as tests of normality and adjustment for multiple comparisons |
| ☐ | ☒ | A full description of the statistical parameters including central tendency (e.g. means) or other basic estimates (e.g. regression coefficient) AND variation (e.g. standard deviation) or associated estimates of uncertainty (e.g. confidence intervals) |
| ☐ | ☒ | For null hypothesis testing, the test statistic (e.g. *F*, *t*, *r*) with confidence intervals, effect sizes, degrees of freedom and *P* value noted<br>*Give P values as exact values whenever suitable.* |
| ☒ | ☐ | For Bayesian analysis, information on the choice of priors and Markov chain Monte Carlo settings |
| ☒ | ☐ | For hierarchical and complex designs, identification of the appropriate level for tests and full reporting of outcomes |
| ☒ | ☐ | Estimates of effect sizes (e.g. Cohen's *d*, Pearson's *r*), indicating how they were calculated |

*Our web collection on statistics for biologists contains articles on many of the points above.*

## Software and code

Policy information about availability of computer code

| | |
|---|---|
| Data collection | UNICORN 7.3 software was used to collect FPLC data. Octet BLI Discovery 12.2.2.20 software was used to collect the bio-layer interferometry data. Gen5 3.08.01 software was used to collect the ELISA data.7500 Fast Software v2.3 was used to collect the Differential scanning fluorimetry data. |
| Data analysis | - GraphPad Prism 9.3.1.<br>- Octet Analysis Studio 12.2.2.26 Software.<br>- XDS Package (version January 10, 2022), Phenix 1.16 and 1.20 (includes Phaser, AutoBuild, and Phenix.refine), Coot 0.9.8.1 and PyMol 2.5.1 curated by SBGrid.<br>- Protein Thermal Shift software v 1.4 |

For manuscripts utilizing custom algorithms or software that are central to the research but not yet described in published literature, software must be made available to editors and reviewers. We strongly encourage code deposition in a community repository (e.g. GitHub). See the Nature Portfolio guidelines for submitting code & software for further information.



March 2021

## Data

Policy information about availability of data

All manuscripts must include a data availability statement. This statement should provide the following information, where applicable:

- Accession codes, unique identifiers, or web links for publicly available datasets
- A description of any restrictions on data availability
- For clinical datasets or third party data, please ensure that the statement adheres to our policy

All data generated or analyzed during this study are included in this published article, source data file and supplementary information files. Atomic coordinates and structure factors have been deposited in the Protein Data Bank with PDB IDs 8GID, 8GIE, and 8GIF. Source data are provided with this paper.

## Human research participants

Policy information about studies involving human research participants and Sex and Gender in Research.

| | |
|---|---|
| Reporting on sex and gender | Not applicable |
| Population characteristics | Not applicable |
| Recruitment | Not applicable |
| Ethics oversight | Not applicable |

Note that full information on the approval of the study protocol must also be provided in the manuscript.

# Field-specific reporting

Please select the one below that is the best fit for your research. If you are not sure, read the appropriate sections before making your selection.

☒ Life sciences ☐ Behavioural & social sciences ☐ Ecological, evolutionary & environmental sciences

For a reference copy of the document with all sections, see nature.com/documents/nr-reporting-summary-flat.pdf

# Life sciences study design

All studies must disclose on these points even when the disclosure is negative.

| | |
|---|---|
| Sample size | Sample sizes were chosen based on animal availability and the ability to see trends in the event of one or two outlier animals. The group sizes are sufficient to support the primary conclusion of the manuscript by showing statistically significant improvement in % GIA elicited by SBD1 immunogen compared to AMA1 DI-DII-RON2L complex. |
| Data exclusions | No data points were excluded from statistical analysis. |
| Replication | In vitro assays were independently replicated at least twice to ensure reproducibility, and all replication attempts were successful. Animal studies were not repeated to conform with proper animal usage guidelines. All data from the rat immunogenicity study are reported in the manuscript. |
| Randomization | Animals were randomly assigned to receive the indicated immunizations. |
| Blinding | All groups were blinded for the growth inhibition assay. |

# Reporting for specific materials, systems and methods

We require information from authors about some types of materials, experimental systems and methods used in many studies. Here, indicate whether each material, system or method listed is relevant to your study. If you are not sure if a list item applies to your research, read the appropriate section before selecting a response.

## Materials & experimental systems

| n/a | Involved in the study | |
|---|---|---|
| ☐ | ☒ | Antibodies |
| ☐ | ☒ | Eukaryotic cell lines |
| ☒ | ☐ | Palaeontology and archaeology |
| ☐ | ☒ | Animals and other organisms |
| ☒ | ☐ | Clinical data |
| ☒ | ☐ | Dual use research of concern |

## Methods

| n/a | Involved in the study | |
|---|---|---|
| ☒ | ☐ | ChIP-seq |
| ☒ | ☐ | Flow cytometry |
| ☒ | ☐ | MRI-based neuroimaging |

# Antibodies

Antibodies used

The goat anti-rat antibody conjugated to Horseradish Peroxidase (HRP) (catalog no.: 112-035-071, Polyclonal, Lot: 153607) purchased from Jackson ImmunoResearch was used as a secondary antibody in ELISA. The mouse 6x-His Tag Monoclonal Antibody (catalog no.: 37-2900, Lot: WC318953) purchased from Thermo Fisher Scientific was used as a primary antibody in western blotting. The goat anti-mouse antibody conjugated to HRP (catalog no.: 115-035-164, Polyclonal, Lot: 156577) purchased from Jackson ImmunoResearch was used as a secondary antibody in western blotting. The immunoglobulin new antigen receptor (IgNAR) 14I-1 was expressed in HEK293 cells (Expi293F™ cells).

Validation

The Immunoglobulin new antigen receptor (IgNAR) 14I-1 was validated in this study by sequencing, ELISA, and Biolayer interferometry. The goat anti-rat antibody conjugated to Horseradish Peroxidase (HRP) (catalog no.: 112-035-071, Polyclonal, Lot: 153607) purchased from Jackson ImmunoResearch  was validated in numerous previous scientific publications and by the manufacturer. The mouse 6x-His Tag Monoclonal Antibody (catalog no.: 37-2900, Lot: WC318953) purchased from Thermo Fisher Scientific was validated in numerous previous scientific publications and by the manufacturer. The goat anti-mouse antibody conjugated to HRP (catalog no.: 115-035-164, Polyclonal, Lot: 156577) purchased from Jackson ImmunoResearch was validated in numerous previous scientific publications and by the manufacturer.

# Eukaryotic cell lines

Policy information about cell lines and Sex and Gender in Research

Cell line source(s)

Expi293F™ cells were purchased from Thermo Fisher Scientific (cat#A14527).

Authentication

Expi293F™ cells were authenticated for Viability and Mycoplasma (Mycoplasma qPCR Assay) by Thermo Fisher Scientific. Expi293F™ cells were more than 90 % viable and negative for Mycoplasma.

Mycoplasma contamination

The cells were not tested for Mycoplasma contamination. No previous case of contamination was ever detected in our laboratory.

Commonly misidentified lines
(See ICLAC register)

No commonly misidentified lines were used.

# Animals and other research organisms

Policy information about studies involving animals; ARRIVE guidelines recommended for reporting animal research, and Sex and Gender in Research

Laboratory animals

12-14-week-old CD (Sprague Dawley) IGS female rats, Crl:CD(SD) (Charles River Laboratories) were used for immunogenicity study.

Wild animals

The study did not involve wild animals.

Reporting on sex

Sex was not considered in the study design.

Field-collected samples

The study did not use field-collected samples

Ethics oversight

Rat immunogenicity study were performed in an American Association for Accreditation of Laboratory Animal Care-accredited facility under the guidelines and approval of the Institutional Animal Care and Use Committee at the National Institutes of Health.

Note that full information on the approval of the study protocol must also be provided in the manuscript.



March 2021
